# The two *C. elegans* class VI myosins, SPE-15/HUM-3 and HUM-8, share similar motor properties, but have distinct developmental and tissue expression patterns

**DOI:** 10.3389/fphys.2024.1368054

**Published:** 2024-04-10

**Authors:** Ranya Behbehani, Chloe Johnson, Alexander J. Holmes, Matthew J. Gratian, Daniel P. Mulvihill, Folma Buss

**Affiliations:** ^1^ Cambridge Institute for Medical Research, University of Cambridge, Cambridge, United Kingdom; ^2^ School of Biosciences, University of Kent, Canterbury, United Kingdom

**Keywords:** MYO6, SPE-15/HUM-3, HUM-8, actin, *C. elegans*

## Abstract

Myosins of class VI move toward the minus-end of actin filaments and play vital roles in cellular processes such as endocytosis, autophagy, protein secretion, and the regulation of actin filament dynamics. In contrast to the majority of metazoan organisms examined to date which contain a single MYO6 gene, *C. elegans*, possesses two MYO6 homologues, SPE-15/HUM-3 and HUM-8. Through a combination of *in vitro* biochemical/biophysical analysis and cellular assays, we confirmed that both SPE-15/HUM-3 and HUM-8 exhibit reverse directionality, velocities, and ATPase activity similar to human MYO6. Our characterization also revealed that unlike SPE-15/HUM-3, HUM-8 is expressed as two distinct splice isoforms, one with an additional unique 14 amino acid insert in the cargo-binding domain. While lipid and adaptor binding sites are conserved in SPE-15/HUM-3 and HUM-8, this conservation does not enable recruitment to endosomes in mammalian cells. Finally, we performed super-resolution confocal imaging on transgenic worms expressing either mNeonGreen SPE-15/HUM-3 or wrmScarlet HUM-8. Our results show a clear distinction in tissue distribution between SPE-15/HUM-3 and HUM-8. While SPE-15/HUM-3 exhibited specific expression in the gonads and neuronal tissue in the head, HUM-8 was exclusively localized in the intestinal epithelium. Overall, these findings align with the established tissue distributions and localizations of human MYO6.

## Introduction

Unconventional myosins constitute a superfamily of motor proteins that translocate along actin filaments and play various roles in diverse cellular processes such as muscle contraction, intracellular trafficking, cell motility, endocytosis, exocytosis, and cytokinesis (Sellers, 2000; Krendel and Mooseker, 2005; Hartman and Spudich, 2012). In humans, a total of 40 myosin genes have been identified that can be grouped into 12 classes based on their distinctive domain structure and organization (Odronitz and Kollmar, 2007). Myosin motors are widely expressed across different tissues and are essential for normal cellular activities.

*Caenorhabditis elegans* is a species of free-living, non-parasitic nematode commonly used as a model organism, being studied extensively with respect to development and genetics, cell lineage, and in work surrounding ageing and human disease (Brenner, 1974). Its genome contains approximately 19,985 protein-coding genes, 38% of which have human orthologues (Wormbase data release WS282, 2021) (Celegans Sequencing Consortium, 1998). In the nematode, nine conventional myosins of class II and seven unconventional myosins have been identified: these include two members of class I, one class V myosin, two class VI myosins, one class VII myosin, one class IX myosin and one myosin of class XII (Baker and Titus, 1997; Johnson et al., 2022; Kollmar and Muhlhausen, 2017). Except for class XII, which is exclusive to *C. elegans*, all other myosin motors show a high degree of similarity to their human paralogues. Interestingly, unlike humans which have only a single myosin VI protein, *C. elegans* possesses two distinct myosins belonging to this class, namely, HUM-3 and HUM-8 (Baker and Titus, 1997). HUM-3 is also called SPE-15 (defective spermatogenesis-15) and so the name SPE-15/HUM-3 will be used in this study (L'Hernault et al., 1988).

In vertebrates, myosins of class VI move towards the minus-end of actin filaments, in direct contrast to all other myosins characterised to date which move in the opposite direction (Wells et al., 1999). Human myosin VI (MYO6) is involved in a range of specific cellular functions such as endocytosis, receptor trafficking, protein secretion and autophagy (Buss et al., 2001; Morris et al., 2002; Aschenbrenner et al., 2003; Warner et al., 2003; Osterweil et al., 2005; Chibalina et al., 2007; Tumbarello et al., 2012; Masters et al., 2017). Loss of these functions, as observed in MYO6-deficient Snell’s waltzer mice and in humans with mutations in the MYO6 gene, contributes to various disease phenotypes, including deafness, astrogliosis, proteinuria, and hypertrophic cardiomyopathy (Avraham et al., 1995; Avraham et al., 1997; Melchionda et al., 2001; Mohiddin et al., 2004; Arden et al., 2016). Interestingly, MYO6 overexpression is commonly observed in diverse cancers, including prostate and ovarian cancer (Yoshida et al., 2004; Dunn et al., 2006).

The diverse cellular roles and phenotypic outcomes associated with MYO6 arise from its interactions with multiple cargo adaptors that play a critical role in directing the motor to its appropriate cellular location and function. These MYO6 adaptor proteins bind either to the RRL or the WWY motifs, located in distinct subdomains of the unique C-terminal cargo-binding tail (Bunn et al., 1999; Sahlender et al., 2005; Chibalina et al., 2007; Morriswood et al., 2007; Spudich et al., 2007; Finan et al., 2011). In addition, the tail contains a phosphatidylinositol 4,5-bisphosphate (PIP2) binding motif, important for recruitment of the motor to intracellular membranes, as well as two distinct ubiquitin-binding sites–a motif interacting with ubiquitin (MIU) and a MYO6 ubiquitin-binding domain (MyUb) (Penengo et al., 2006; Spudich et al., 2007; He et al., 2016). The cargo-binding tail of human MYO6 undergoes alternative splicing of two inserts: the small insert (SI, adding nine residues) and the large insert (LI, adding 31 residues). This gives rise to four splice isoforms containing the SI, LI, both, or neither. The different splice variants of MYO6 have differential interaction networks and binding partners, generating substantial potential for diversity in MYO6 function (de Jonge et al., 2019). As such, the variants can be found in different cell types and tissues. The LI isoform, for example, is specifically expressed in polarised epithelial cells containing microvilli at their apical domain, whereas the SI and no insert (NI) isoforms are expressed in cells lacking apical microvilli (Buss et al., 2001; Morris et al., 2002; Ameen and Apodaca, 2007; Wollscheid et al., 2016).

MYO6 plays a central role at several steps along the endocytic and exocytic pathways. The LI variant, for example, is involved in cell-surface receptor trafficking in epithelial cells (Buss et al., 2001; Wollsheid et al., 20,160). The role of MYO6 in clathrin-mediated endocytosis involves Dab2, an endocytic adaptor protein (Morris et al., 2002). The NI isoform, on the other hand, localises to Rab5-and APPL1-positive peripheral early endosomes, and is required for the movement of these endosomes through the actin-rich cell cortex away from the plasma membrane (Aschenbrenner et al., 2003; Chibalina et al., 2007; Masters et al., 2017). Recruitment of MYO6 to APPL1-positive endosomes involves the adaptor proteins GIPC and TOM1L1/2 (Buss et al., 1998; Tumbarello et al., 2012). The role of MYO6 in the autophagic pathway during autophagosome maturation is mediated through its cargo adaptor proteins optineurin (OPTN), TAX1 binding protein 1 (TAX1BP1), and nuclear dot protein 52 (NDP52), which also function as selective autophagy receptors (Tumbarello et al., 2012).

In contrast to the well-characterised human MYO6, the two MYO6 homologues expressed in *C. elegans*, SPE-15/HUM-3 and HUM-8, have not been extensively studied and very limited information is available regarding their function, interactome, or biophysical properties. Although there is no published literature specifically on HUM-8, it was found to localise within the mammalian midbody, a structure known to contain proteins involved in cytokinesis (Skop et al., 2004). Interestingly, MYO6 has also been shown to play a role in membrane delivery during mammalian cytokinesis, highlighting a potentially evolutionarily conserved function during cell division for HUM-8 (Arden et al., 2007).

A role for the second MYO6 homologue, SPE-15/HUM-3, has been established in spermiogenesis. In *C. elegans*, spermatogenesis leads to the formation of ameboid spermatozoa, and involves the asymmetric partitioning of cellular material. During spermatid differentiation, spermatids shed unwanted cellular material in the form of an acellular remnant, the residual body (RB), later detaching from this to become motile spermatozoa. A *SPE-15/HUM-3* null deletion mutant produces gross cytological defects in the morphology of the RB and budding spermatids, which typically fail to form spermatozoa (Kelleher et al., 2000). This is consistent with the observation that *SPE-15/HUM-3* mutant hermaphrodites are almost completely self-sterile (L'Hernault et al., 1988). In addition to this, SPE-15/HUM-3 appears to play a role in the final cytokinetic step during spermatid budding. Interestingly, this process is dependent on *C. elegans* GIPC-1 and GIPC-2, homologues of the human GIPC protein family, a group of known MYO6 adaptor proteins (Hu et al., 2019).

*C. elegans* is a well-established *in vivo* model system that has emerged as an important tool in pharmacological drug discovery. Myosin motors are established druggable targets and, to date, several allosteric effectors of different classes of myosins have been identified. To make potential use of *C. elegans* in a pharmacological screen for modulators of MYO6 activity, we require a more complete understanding of the cellular, biochemical, and biophysical characteristics of the *C. elegans* MYO6 homologues. Beyond the role of SPE-15/HUM-3 in spermatogenesis, very little is known about the overall functions of SPE-15/HUM-3 and HUM-8 in different nematode tissues. Indeed, it is unclear how the roles of SPE-15/HUM-3 and HUM-8 differ, and how they compare to human MYO6 in terms of functional diversification. This study therefore sought to uncover the cellular characteristics of SPE-15/HUM-3 and HUM-8 both endogenously in *C. elegans*, and in mammalian cell systems, to provide a starting point for a detailed understanding of these MYO6 homologues.

Our findings indicate that there are no significant differences in the biophysical motor characteristics of SPE-15/HUM-3 and HUM-8 concerning their actin gliding velocity and ATPase activity. These attributes closely resemble those of human MYO6 including the reverse directionality along actin filaments, which we confirmed using a filopodia tip recruitment assay. Notably, while crucial adaptor and lipid binding sites are conserved in the cargo-binding tail domain of SPE-15/HUM-3 and HUM-8, this conservation does not facilitate recruitment to peripheral endosomes underneath the plasma membrane, where human MYO6 and the *Drosophila* homologue of MYO6, *Jaguar*, can be found. With the tail domain of human MYO6 known to be a site of alternative splicing, we also sought to uncover whether SPE-15/HUM-3 and HUM-8 undergo similar splicing patterns. Our results show that HUM-8 undergoes alternative splicing, resulting in two isoforms with one containing a unique 14 amino acid insert in the cargo binding domain, whilst SPE-15/HUM-3 is expressed as a single splice isoform. Finally, extensive super-resolution confocal imaging performed on transgenic worms highlights a distinct tissue distribution of these two MYO6 homologues in *C. elegans*. Whereas SPE-15/HUM-3 is specifically expressed in the gonads and neuronal tissue, HUM-8 can almost exclusively be found in the intestinal epithelium. Interestingly, this coincides with the known tissue distributions and localisations of MYO6 in mammalian cells.

## Results

### SPE-15/HUM-3 and HUM-8 have similar biophysical and structural characteristics compared to human MYO6

The cellular functions of human MYO6 are facilitated by adaptations in its protein structure, binding motifs, splice isoforms and various interaction domains. To determine the conservation of these key features across species, we performed a sequence and structural comparison of SPE-15/HUM-3 and HUM-8 with human MYO6 and *Drosophila* Jaguar. *Drosophila* was chosen here as it is another well-characterised model organism, and its MYO6 homologue has well-established functions that could be used as a reference point. A multiple sequence alignment of SPE-15/HUM-3, HUM-8, Jaguar (isoform B) and human MYO6 (isoform three containing both the LI and SI) revealed a 48% sequence identity between SPE-15/HUM-3 and human MYO6 and a 45% identity between HUM-8 and MYO6 (Supplementary Figure S1). Interestingly, Jaguar is more similar in sequence to human MYO6 (51%) than it is to either SPE-15/HUM-3 (43%) or HUM-8 (39%). The two myosins from *C. elegans* are only 63% identical, highlighting the potential for functional variation between SPE-15/HUM-3 and HUM-8. The overall level of conservation between SPE-15/HUM-3, HUM-8, human MYO6 and Jaguar confirms the evolutionary relationship between these four different myosins of class VI.

**FIGURE 1:**
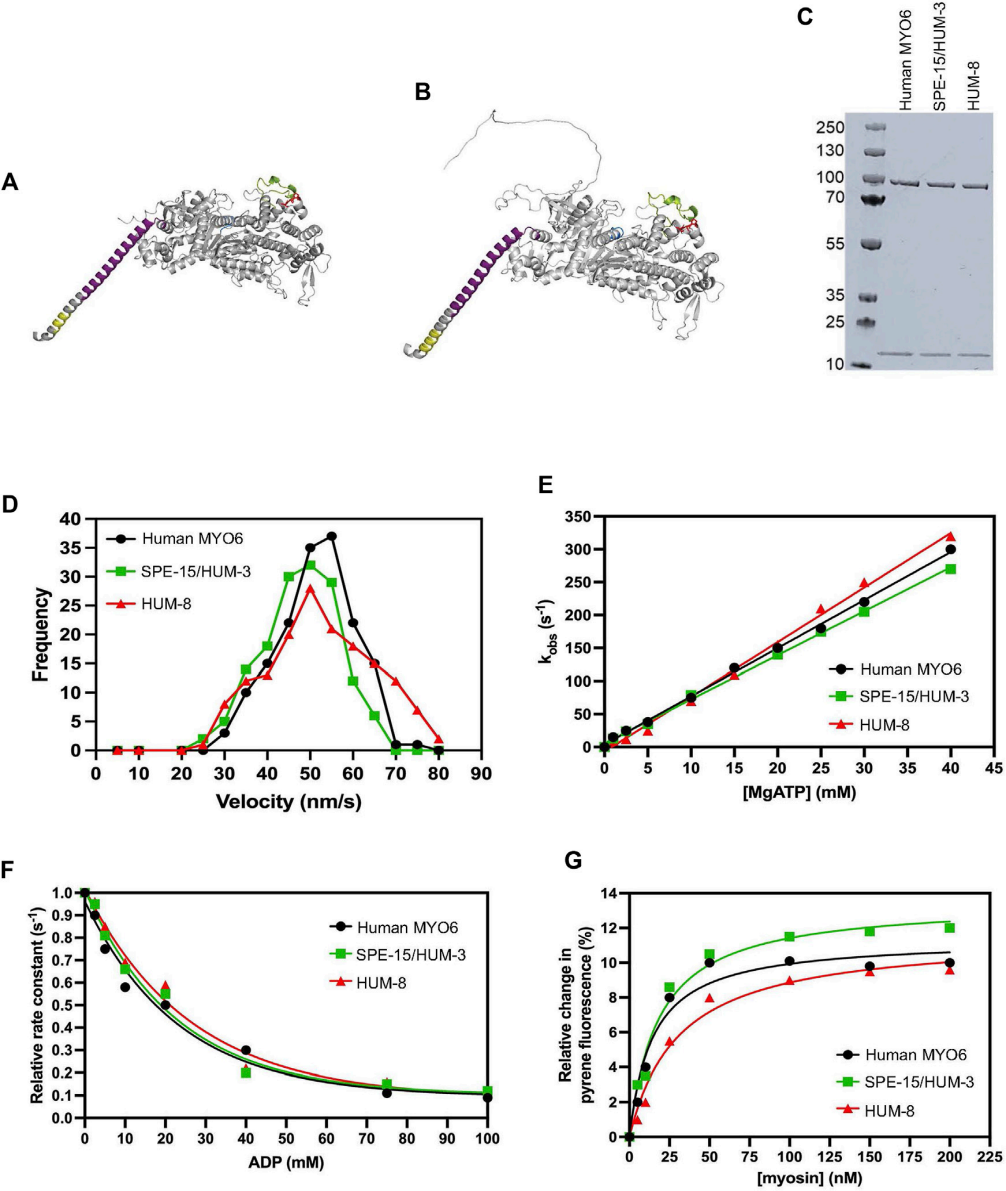
Biophysical properties of SPE-15/HUM-3 and HUM-8 compared to human MYO6. Predicted SPE-15/HUM-3 **(A)** and HUM-8 **(B)** *AlphaFold* structures of the motor and neck region. The P-loop is shown in blue, insert-1 in green, insert-2 in purple and IQ motif in yellow. **(C)** SDS-PAGE of recombinant SPE-15/HUM-3 and HUM-8 motor neck domain expressed and purified from *ExpiSF9* cells. **(D)** Actin-gliding velocity of rhodamine-phalloidin labelled actin filaments of human MYO6 (black), SPE-15/HUM-3 (green), and HUM-8 (red) motor domains as determined by *in vitro* motility assays in at least three independent experiments. The histograms show typical distributions of actin filament velocities on myosin-decorated surfaces. The mean velocity for human MYO6 was 55 ± 2.3 nm/s, 51 ± 3.3 nm/s for SPE-15/HUM-3 and 49 ± 5.6 nm/s for HUM-8 as calculated using a Gaussian fit. No statistical difference was observed between these values. **(E)** The effect of ATP concentration on the observed rate constant for ATP-induced dissociation of pyrene-actin. myosin for human MYO6 (black), SPE-15/HUM-3 (green) and HUM-8 (red). The gradient generates a second-order rate constant of ATP binding. **(F)** Plot of relative observed rate constant dependence on ADP concentration for the ATP-induced dissociation of pyrene-actin. myosin from human MYO6 (black), SPE-15/HUM-3 (green) and HUM-8 (red). **(G)** Fluorescence amplitude plotted as a function of myosin concentration can be described by a quadratic fit resulting in the affinity for actin of human MYO6 (black), SPE-15/HUM-3 (green) and HUM-8 (red).

Both SPE-15/HUM-3 and HUM-8 have N-terminal extensions just prior to the start of the motor domain (8 amino acids in SPE-15/HUM-3, 73 residues in HUM-8) not found in either human MYO6 or Jaguar (Figures 1A,B). The 73 amino acid extension in HUM-8 appears in the AlphaFold model as a long extended loop without any obvious secondary structure and a very low confidence score. Within the SPE-15/HUM-3 and HUM-8 motor domain and neck region, we found conservation of sequences functionally important for myosin motors, including the characteristic GESGAGKT sequence of the ATP-binding P-loop and a single IQ calmodulin-binding motif (as in human MYO6), as well as key regions that define myosins of class VI: insert-1 (involved in regulation of nucleotide binding) and insert-2 (the ‘reverse gear’, responsible for reverse directionality) (Menetrey et al., 2005; Bryant et al., 2007). Predicted SPE-15/HUM-3 and HUM-8 AlphaFold structures have the same overall motor domain organisation compared to structures of human MYO6 without any indication of major structural changes (see Figure 1 A, B) (Jumper et al., 2021).

We next analysed the biophysical motor properties using *in vitro* motility assays and stopped-flow spectroscopy. The SPE-15/HUM-3 and HUM-8 motor and neck domains were co-expressed with calmodulin and purified from SF9 cells (Figure 1C). To determine the ATPase activity of SPE-15/HUM-3 and HUM-8, and to study the interaction of these myosins with F-actin, we measured the translocation speed of rhodamine-phalloidin labelled actin filaments by surface-immobilised SPE-15/HUM-3, HUM-8 or human MYO6 (Figure 1D). Our results show that SPE-15/HUM-3 and HUM-8 have similar average velocities compared to human MYO6, with gliding speeds of 52 ± 8 nm/s, 49 ± 6 nm/s and 55 ± 9 nm/s, respectively.

All myosins are united by a characteristic cyclical interaction with actin driven by ATP hydrolysis, which produces mechanical movement as a result of structural changes in the motor (Preller and Manstein, 2013). To determine if any part of the mechanochemical ATPase cycle is altered in SPE-15/HUM-3 or HUM-8 compared to human MYO6, we measured their fast reaction kinetics using stopped-flow spectroscopy. This assay measures the fluorescence of pyrene-labelled actin which is quenched when complexed to a myosin. The myosin is released from actin after ATP-binding, resulting in an increase in pyrene fluorescence. This set-up was used to measure the second order rate constant of ATP-induced dissociation of an actin.myosin complex. All three proteins follow single exponentials with no lag phase. The dependence of the observed rate constant on ATP concentration is shown in Figure 1E. The gradients of these plots were used to determine the apparent second order rate constants for ATP binding to actin.myosin, which for the three proteins were as follows; human MYO6: 5 ± 0.4 mM^-1^s^-1^, SPE-15/HUM-3: 9 ± 0.8 mM^-1^s^-1^ and HUM-8: 3 ± 0.5 mM^-1^s^-1^. Although overall very similar, the slight differences may indicate altered nucleotide affinities for the three motors. To test this further, we compared the affinity of SPE-15/HUM-3, HUM-8 or MYO6 for ADP in the presence of actin using a competition assay. In this set up, the actin.myosin complex is preincubated with varying concentrations of ADP, which led to a competition in binding between the ATP and ADP. The resulting observed rate can be plotted as a function of ADP concentration, which has a hyperbolic dependence (Figure 1F). SPE-15/HUM-3 and HUM-8 have slightly weaker affinities for ADP (18 ± 4 mM and 20 ± 2 mM respectively) compared to human MYO6 (6 ± 0.8 mM). These parameters indicate small modulations in motor activity with ATP and ADP.

Finally, to determine the affinity of SPE-15/HUM-3 and HUM-8 to actin, the ATP-induced dissociation reaction was performed with varying myosin concentrations. At higher concentrations of myosin, an increasing amount of pyrene will be quenched at the beginning of the measurement, leading to an increase in fluorescence amplitude (Figure 1G). The plotted relative amplitude as a function of myosin concentration can be described by a quadratic equation, resulting in the actin affinity. The affinities for human MYO6 and SPE-15/HUM-3 were similar, with values of 15 ± 3 and 13 ± 5 nM respectively. HUM-8 has a slightly weaker affinity value of 23 ± 5 nM, although this figure still being in the sub-nanomolar range is unlikely to impact the function of the motor. Overall, these results demonstrate that both SPE-15/HUM-3 and HUM-8 have kinetic characteristics similar to human MYO6, which are typical of a class VI myosin.

### SPE-15/HUM-3 and HUM-8 do not accumulate at the plus ends of actin filaments

**FIGURE 2:**
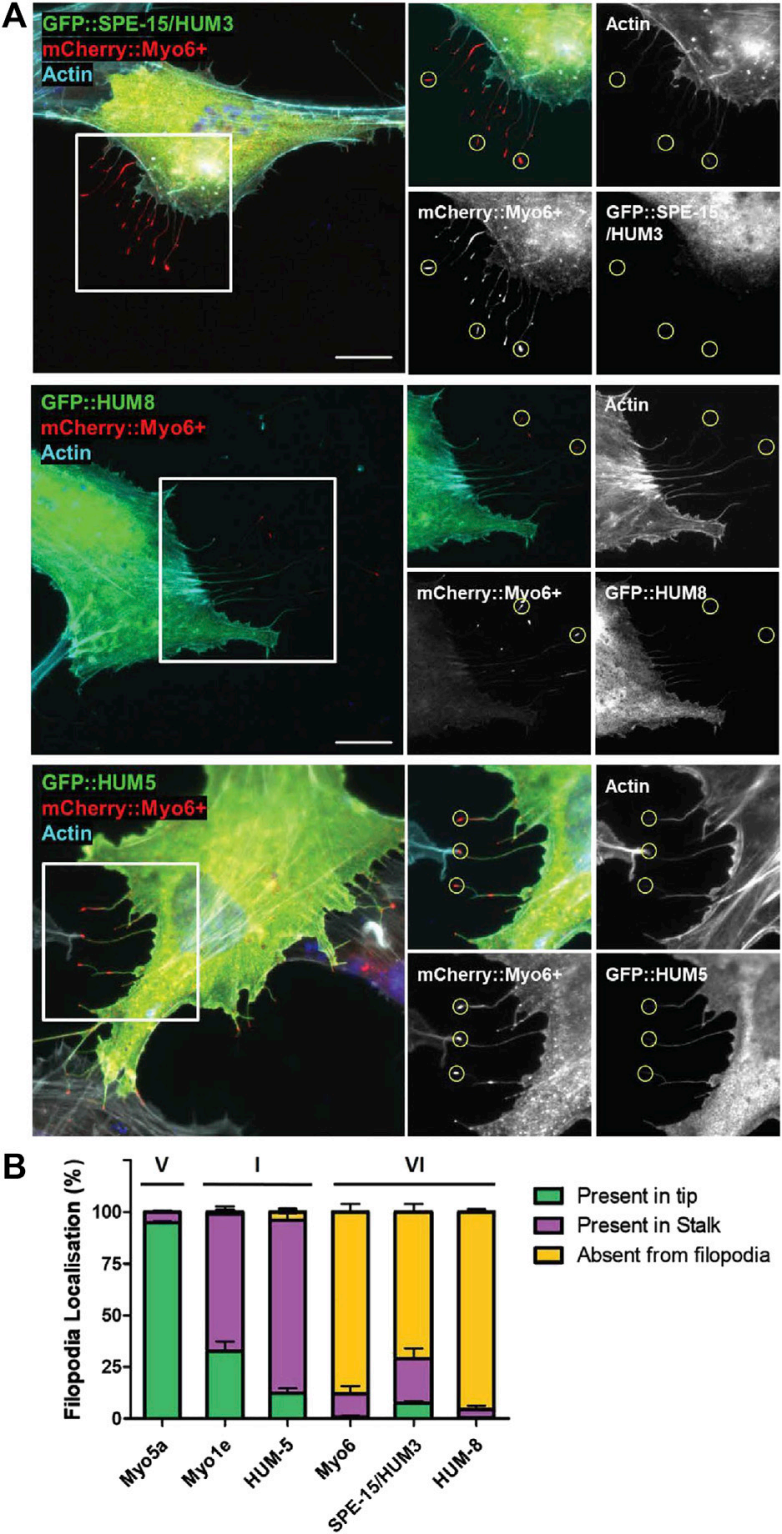
Localisation of SPE-15/HUM-3 and HUM-8 in filopodia as a measure of their directionality. **(A)** RPE cells co-transfected with GFP::SPE-15/HUM-3 (top), GFP::HUM-8 (middle) or GFP::HUM-5 (bottom) alongside mCherry:Myo6+. Actin filaments are labelled using Phalloidin-647 (cyan), Nuclei are labelled using Hoechst (blue). Yellow circles indicate examples of representative filopodia tips. Scale bar, 10 μm. **(B)** Quantification of GFP:myosin localisation in filopodia of RPE cells. 100 filopodia were counted per coverslip and assigned either ‘present in tip’, ‘present in stalk’ or ‘absent from filopodia’ localisation by eye from n = 3 independent experiments per condition. Error bars indicate SEM.

Both myosins, SPE-15/HUM-3 and HUM-8, contain the insert-2 in the converter region that repositions the lever arm to trigger reverse movement towards the minus-end of actin filaments (Supplementary Figure S1) (Wells et al., 1999; Menetrey et al., 2005; Bryant et al., 2007). To test whether SPE-15/HUM-3 and HUM-8 indeed share the reverse directionality with other myosins of class VI, we visualised the localisation of SPE-15/HUM-3 and HUM-8 in filopodia at the cell surface of RPE cells. To induce the formation of these filopodia, we transfected into RPE cells a mutant form of human MYO6, termed MYO6+, which is a genetically engineered plus-end directed MYO6 whose neck region, including insert-2, is replaced with the lever arm of MYO5, a plus-end tracking myosin (Masters and Buss, 2017). The mutant MYO6 induces the formation and accumulates at the tips of filopodia, thereby marking the plus-ends of actin filament bundles inside these plasma membrane protrusions. To test if SPE-15/HUM-3 and HUM-8 are indeed minus-end directed myosins, they were co-expressed together with MYO6+ in RPE cells. In addition to SPE-15/HUM-3, HUM-8 (Figure 2A) and human wild-type MYO6, we also tested the localisation of three plus-end directed myosins within filopodia: human MYO5, human MYO1E and HUM-5, the *C. elegans* homologue of a class I myosin. We subsequently quantified the localisations of the six myosins, which were either present in filopodia tips (green), along the length of the filopodia stalk (purple), or absent from filopodia altogether (yellow). The results summarized in Figure 2B indicate that the three myosins of class VI predominantly accumulate in the cytosol, and none are found in filopodia. In contrast, human MYO5A shows almost complete accumulation at the tips of filopodia, whilst the two myosins of class I are present along the length of filopodia with a modest concentration at the tips. These results may indicate that MYO5A as a processive, dimeric motor is able to maintain its position at the fast growing plus ends of actin filaments, while the slower, monomeric myosins of class I are moved away from filopodia tips *via* actin treadmilling. In summary, these experiments strongly suggest that SPE-15/HUM-3 and HUM-8 are not able to move towards the plus-end of actin filaments, confirming their classification as reverse motors.

### Two splice variants of HUM-8 are expressed in *C. elegans*

**FIGURE 3:**
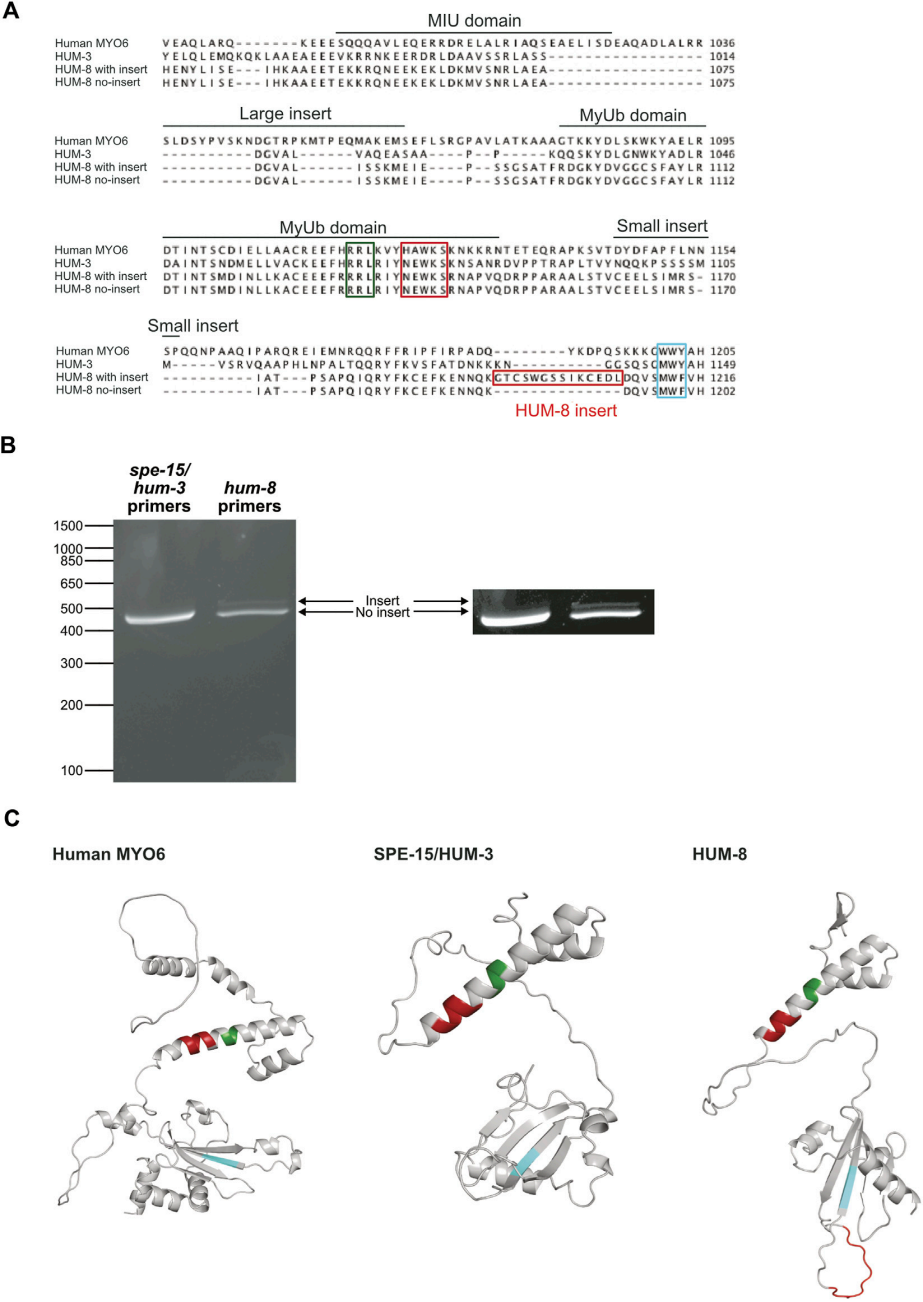
Two splice variants of HUM-8 are expressed in adult worms. **(A)** Multiple sequence alignment of the CBDs of SPE-15/HUM-3, HUM-8 and human MYO6 highlighting key regions for cargo-binding identified in MYO6 such as the RRL-motif (green), HAWKS-motif (dark red), and WWY-sequence (blue). The alignment indicates a 12 bp region in HUM-8 (HUM-8 insert, red) that is uniquely present in one HUM-8 isoform. **(B)** DNA electrophoresis gel of PCR reactions amplifying the CBDs of SPE-15/HUM-3 and HUM-8 directly from purified worm cDNA. Only a single band (468 bp) is present for SPE-15/HUM-3, indicating a single splice variant. Two bands are present in the HUM-8 lane (one at 444 bp and the other slightly larger), indicating two splice variants. The image on the right is modified for enhanced contrast to aid visualisation of the second HUM-8 band. **(C)** *AlphaFold* predicted structure of the CBDs of human MYO6 (left) alongside SPE-15/HUM-3 (middle) and HUM-8 (right), with the cargo-binding motifs outlined in **(A)** highlighted in colour. The unique HUM-8 insert, found on an extended loop, is also shown here in red.

Our earlier sequence alignments (Supplementary Figure S1) indicated that neither SPE-15/HUM-3 nor HUM-8 contain sequences corresponding to the human LI or SI. The alternative splicing of human MYO6 gives rise to four isoforms with functional variation resulting from differences in their binding interactions with adaptor proteins. To determine whether SPE-15/HUM-3 and HUM-8 are similarly alternatively spliced, using PCR we amplified the CBD regions spanning the MIU domain and ending in the WWY motif, which in human MYO6 encompasses both the SI and the LI (highlighted in Figure 3A). The PCR products were run on a DNA agarose gel and show a single band for SPE-15/HUM-3, corresponding to a single splice variant, and two bands for HUM-8, indicating two distinct splice variants (Figure 3B). The three bands were sequenced and aligned to human MYO6, which highlighted a 42 base pair region that was present in just one of the two HUM-8 variants (amino acid sequence highlighted in red box, Figure 3A). Whilst this sequence of HUM-8 containing the additional insert has previously been computationally mapped as a potential HUM-8 isoform on UniProt, we show here that it is indeed expressed in *C. elegans*. No similar amino acid sequence was present in either SPE-15/HUM-3 or human MYO6, indicating a unique alternatively spliced region (GTCSWGSSIKCEDL) in HUM-8 only, giving rise to two potential isoforms. No homology to any known protein motifs was identified within the sequence, suggesting that if HUM-8 function is diversified through its alternative splicing, then this could possibly point to the existence of novel *C. elegans* adaptor-binding motifs.

### SPE-15/HUM-3 and HUM-8 are not recruited to endosomes in mammalian cells

Cargo adaptor proteins mediate the diverse functions of human MYO6 in different cell types and tissues. From the large array of human MYO6 binding partners, only three appear to have orthologues in *C. elegans* or *Drosophila*: TOM1L2 (C07A12.7 in *C. elegans*; CG3529 in *Drosophila*), GIPC (gipc-1/gipc-2 in *C. elegans*; Kermit in *Drosophila*) and Dab2 (dab-1 in *C. elegans*; Disabled in *Drosophila*). Comparing MYO6 binding partner homologues in *C. elegans* and *Drosophila* thus yields the same repertoire of proteins, whilst most other known MYO6 binding partners do not have homologues in either of these organisms. Both *C. elegans* and *Drosophila* show significant conservation in the two cargo adaptor binding sites, the RRL and the WWY motif. Although the binding site for TOM1L2 and Dab2, the WWY motif, is changed to MWF, the second tryptophan, which has been shown to be crucial for cargo adaptor binding, is conserved (Spudich et al., 2007). In contrast, the RRL motif, the binding site for GIPC, is unchanged in SPE-15/HUM-3, HUM-8 and Jaguar.

**FIGURE 4:**
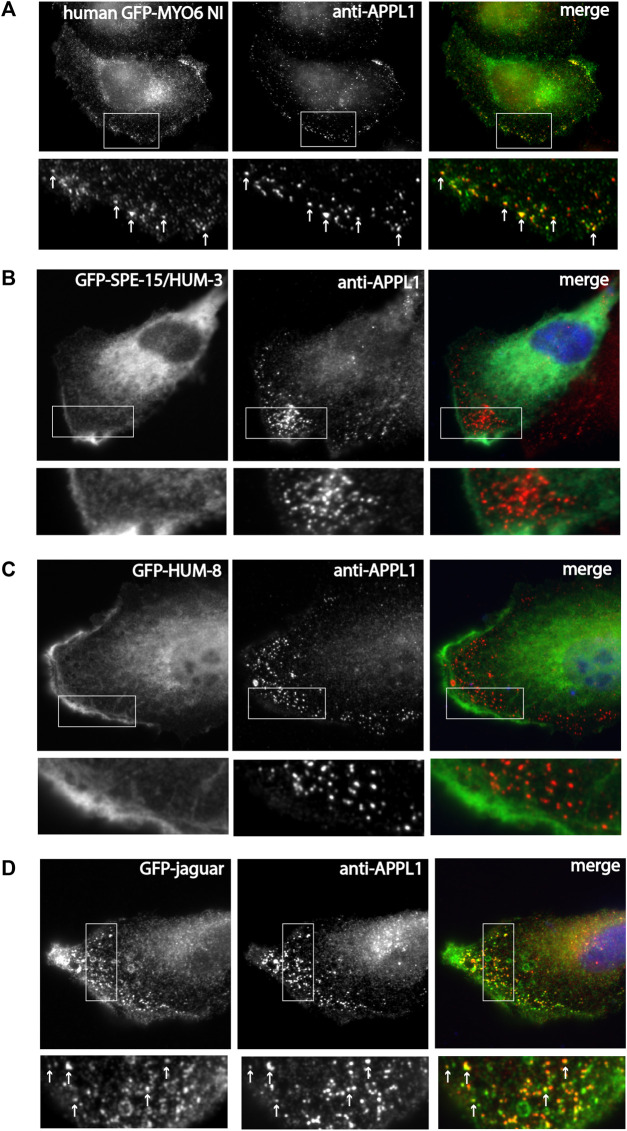
SPE-15/HUM-3 and HUM-8 are not recruited to early endosomes in mammalian cells, in contrast to the Drosophila MYO6 homologue (Jaguar). **(A)** GFP-tagged human MYO6 no insert (NI), **(B)** GFP-SPE-15/HUM-3, **(C)** GFP-HUM-8 and **(D)** GFP-jaguar were expressed in RPE cells and stained with antibodies to APPL1 to label early endosomes. Boxed area is enlarged in the panel below in A-D. White arrows in the boxed area highlight colocalization between the different myosins and APPL1-positive endosomes. While human MYO6 and Dosophila Jaguar are recruited to APPL1-positive endosomes, SPE-15/HUM-3 and HUM-8 show no colocaliation with APPL1. Scale bar, 10 μm.

Since GIPC interacts with APPL1 and is thus important for targeting of human MYO6 to early endosomes in the cell periphery, we next compared the localisation of SPE-15/HUM-3 and HUM-8 as well as Jaguar to human MYO6. SPE-15/HUM-3, HUM-8 (isoform with unique insert), and Jaguar were cloned into the mammalian expression vector pEGFP and, together with the NI isoform of human MYO6, expressed as GFP-fusion proteins in RPE cells. Following fixation, the cells were stained with antibodies to APPL1, the marker protein for early signalling endosomes found in the actin-rich cell cortex. Human NI GFP-MYO6 co-localises precisely with APPL1-positive endosomes in the cell periphery (Figure 4A). In contrast, whilst SPE-15/HUM-3 and HUM-8 are recruited to the plasma membrane at the leading edge of the cell, no colocalisation with APPL1 is evident for the *C. elegans* homologues (Figures 4B,C). Intriguingly, like human MYO6, the expressed *Drosophila* homologue of MYO6, Jaguar, is present on APPL1 labelled endosomes (Figure 4D).

These findings imply that the presence of a conserved RRL motif alone is not solely responsible and sufficient for recruiting these MYO6 variants to early endosomes. It is likely that additional lipid or protein binding motifs that are present in MYO6 and Jaguar but absent from SPE-15/HUM-3 and HUM-8, are required for selective targeting.

### SPE-15/HUM-3 and HUM-8 vary in their developmental expression patterns

**FIGURE 5:**
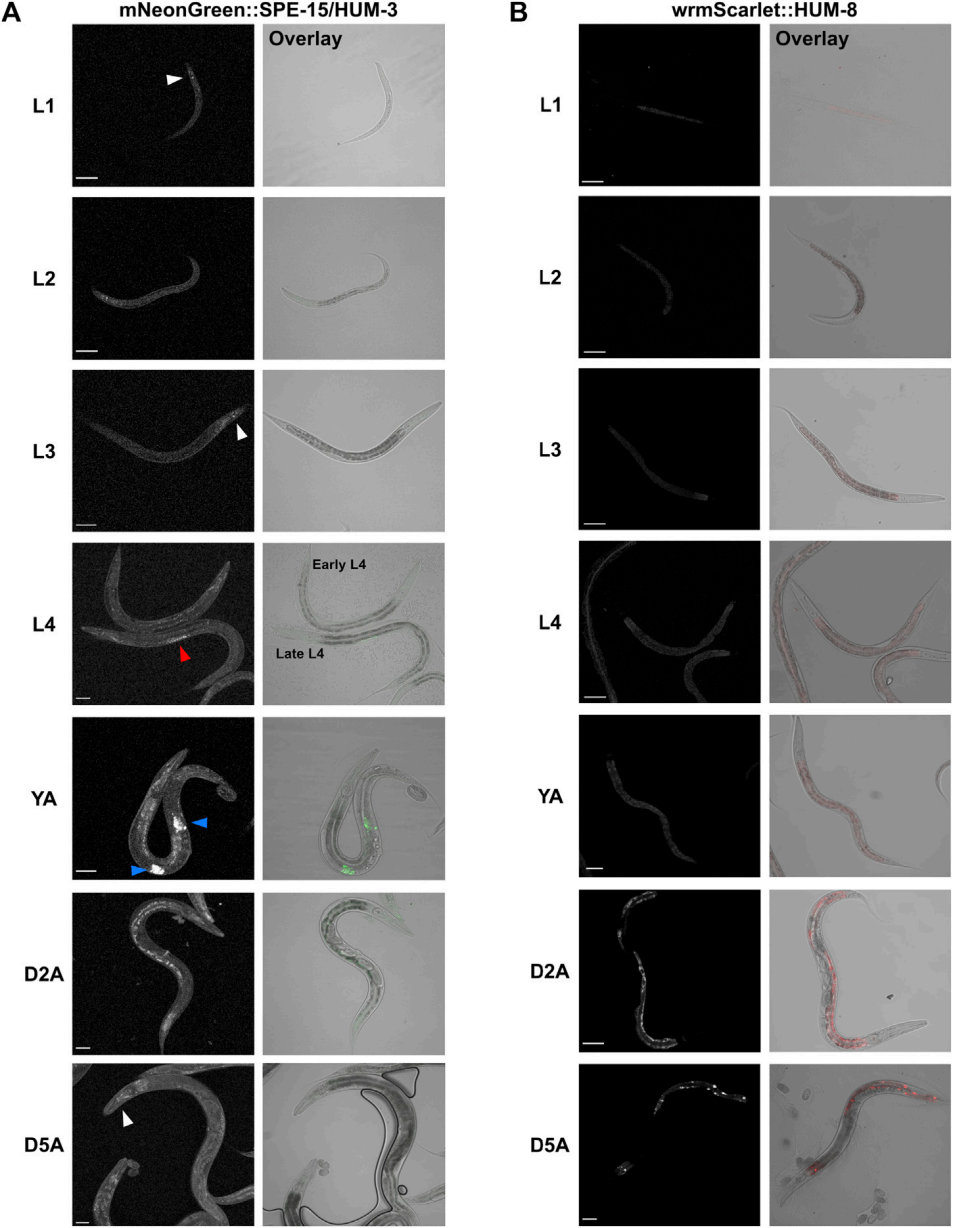
Whole-worm confocal imaging indicates differential developmental and tissue-specific expression of SPE-15/HUM-3 and HUM-8. **(A)** mNeonGreen:SPE-15/HUM-3 and **(B)** wrmScarlet:HUM-8 localisation throughout the larval stages (L1-L4) and into young adulthood (YA) and days 2 (D2A) and 5 (D5A) of adulthood. Images from three independent biological repeats (N = 8–10 animals each) were taken. For each developmental stage, a representative image is shown. White arrows point to SPE-15/HUM-3 localisation in the head, red arrows to localisation throughout the gonads, and blue arrows to a focused localization in each arm of the gonads of the nematode. All images taken at ×20 magnification. Scale bars, 50 µm.

Very little is known about the specific tissue and cellular expression patterns of SPE-15/HUM-3 and HUM-8 across the worm lifespan. To gain insight into their developmental expression as a way to understand their potential functional specialisations, we utilised worms expressing fluorescently-tagged mNeonGreen:SPE-15/HUM-3 and wrmScarlet:HUM-8 (Sunybiotech). Nematodes from every larval stage (L1 to L4), as well as at days 2 and 5 of adulthood, were imaged (Figures 5A,B). Our results show that SPE-15/HUM-3 and HUM-8 are both expressed throughout all nematode developmental stages, but with varying patterns. SPE-15/HUM-3 is expressed in the head throughout the nematode lifespan (white arrows, Figure 5A). At the L4 larval stage, SPE-15/HUM-3 fluorescence is seen throughout the gonads (red arrow, Figure 5A), while at the young adult stage, this gonadal fluorescence pattern is replaced by a distinctive localisation of SPE-15/HUM-3 in each arm of the gonads (blue arrows, Figure 5A). This pattern dissipates after the young adult stage, suggesting a developmental regulation of SPE-15/HUM-3 expression, wherein SPE-15/HUM-3 is upregulated in the gonadal tissues at the L4 stage and subsequently downregulated following young adulthood. In contrast, the expression of HUM-8 is uniform throughout all developmental stages, being enriched almost exclusively in the intestinal epithelium (Figure 5B). There was no observable change in HUM-8 tissue expression at any larval stage, implying a lack of developmental regulation.

## HUM-8 is expressed predominantly within the intestinal epithelium, whereas SPE-15/HUM-3 localises to neuronal tissue and the gonads

**FIGURE 6:**
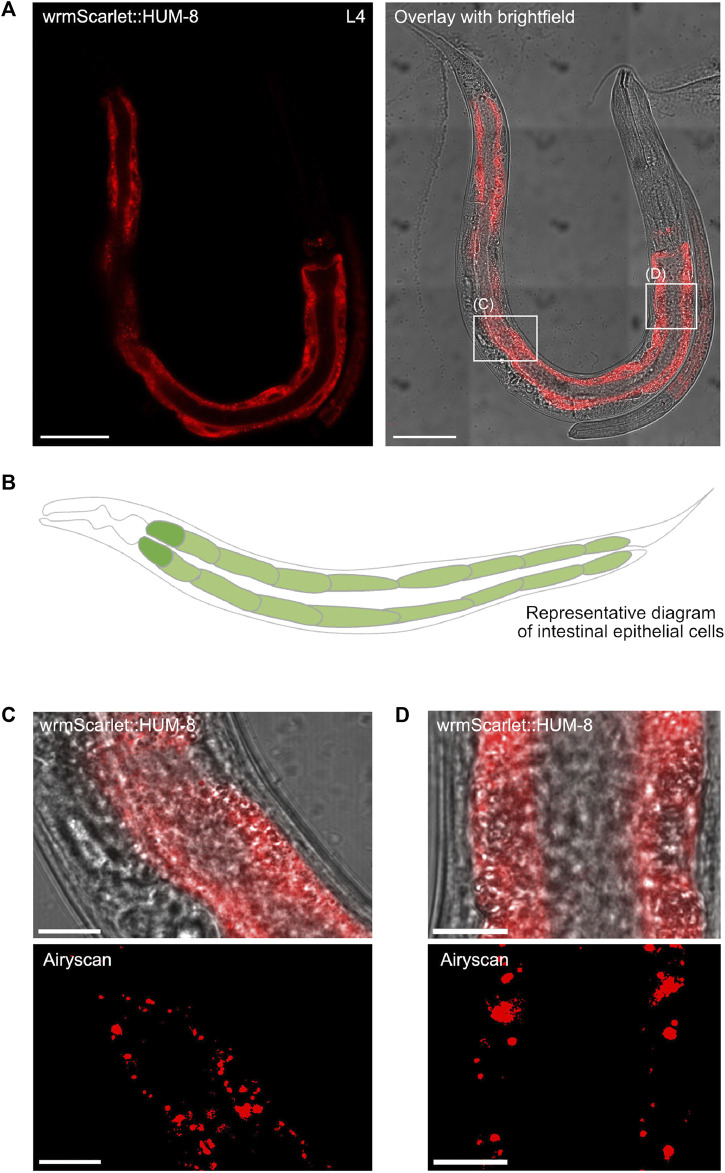
HUM-8 is expressed in the *C. elegans* intestinal epithelium. **(A)** A tile scan confocal image of a young adult worm expressing wrmScarlet:HUM-8 taken at ×63 magnification. Scale bar, 50 µm. **(B)** A representative schematic of the *C. elegans* intestine. Cells are arranged around a hollow core (the intestinal lumen). **(C)** and **(D)** are regular confocal (top) and Airyscan (bottom) images of HUM-8 within the intestinal epithelium from areas outlined in white boxes in **(A)**. Scale bar, 10 µm. **(D)**.

Having determined the developmental expression and distribution of both SPE-15/HUM-3 and HUM-8, we next conducted detailed localisation analysis at high-resolution to determine the tissue and cell-specific distribution of the two myosins. High-magnification tile-scan images of young adult wrmScarlet:HUM-8 worms confirmed that HUM-8 is expressed almost exclusively in the cytosol of intestinal epithelial cells (Figure 6A, dark regions seen where nuclei are excluded from fluorescence). The intestine is composed of large, cuboidal enterocytes that form pairs, each surrounding the intestinal lumen (Figure 6B) (Dimov and Maduro, 2019). HUM-8 is expressed at similar levels in these enterocytes. Super-resolution AiryScan images of the worm shown in Figure 6A (regions of interest highlighted in white boxes) revealed a vesicular localisation pattern of HUM-8 at higher resolution, suggesting that this myosin is localising to vesicles in intestinal cells (Figures 6C,D). Further work, however, will be required to determine the exact nature of these vesicles.

**FIGURE 7:**
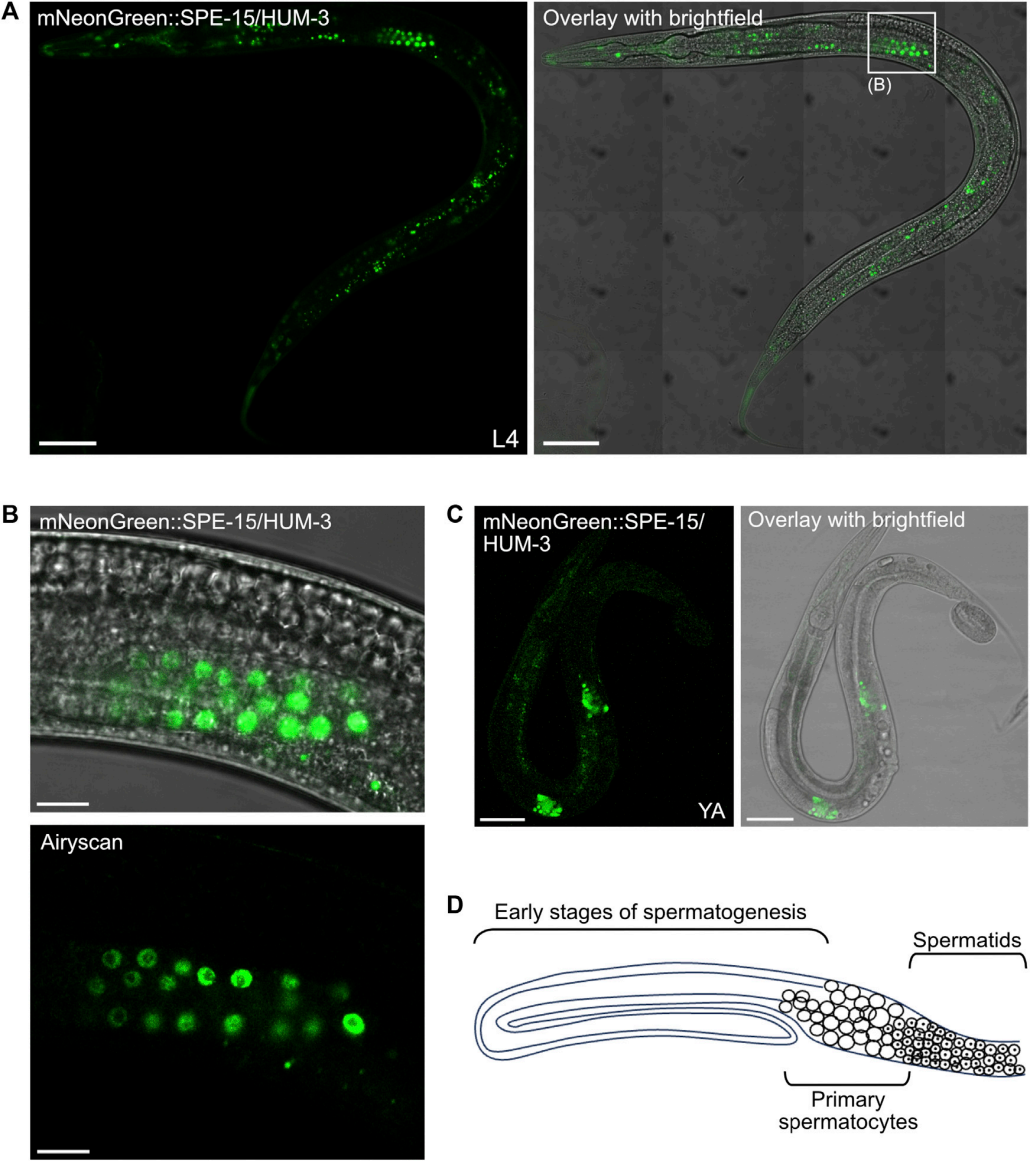
SPE-15/HUM-3 is expressed in the *C. elegans* gonads. **(A)** A tile scan confocal image of an L4 worm expressing mNeonGreen:SPE-15/HUM-3 taken at ×63 magnification. Areas outlined in white boxes are shown at a larger scale in different sections as indicated. Scale bar, 50 µm. **(B)** Regular confocal (top) and Airyscan (bottom) images of SPE-15/HUM-3 localisation in the gonads of an L4 stage worm. Scale bar, 10 µm. **(C)** Regular confocal image of a young adult mNeonGreen:SPE-15/HUM-3 worm showing SPE-15/HUM-3 within the gonads of a young adult worm. Scale bar, 50 µm. **(D)** A representative schematic of one arm of a bilaterally symmetric, two-armed, wild-type hermaphrodite gonad undergoing spermatogenesis. Distal is to the left. The diagram highlights typical organisation of the germline, showing that progression from early spermatogenesis stages into the meiotic stages occurs from distal to proximal. Primary spermatocytes have not yet undergone meiosis I. Following spermatogenesis, mature sperm are stored in a proximal spermatheca, the receptacle in which sperm are stored.

High-magnification tile-scan images of L4 mNeonGreen:SPE-15/HUM-3 worms confirmed the localisation of SPE-15/HUM-3 to three distinct sites: cells within the nematode head, within the tail, and, most prominently, to germ cells in the gonad (white box, Figure 7A). *C. elegans* hermaphrodites exclusively undergo spermatogenesis at the L4 stage. This ceases as the worm develops into an adult, at which point oogenesis begins. A close up of the gonadal regions highlighted in Figure 7B showed SPE-15/HUM-3 expression in a very small subset of developing germ cells towards the vulva and to the proximal end of the L4 gonad. Airyscan images of this region shows that, in these cells, SPE-15/HUM-3 is concentrated in the cytoplasm around the nucleus (Figure 7B). In the young adult stage, as hermaphrodites transition from spermatogenesis to oogenesis, residual expression of SPE-15/HUM-3 is restricted to two regions either side of the vulva (two bright areas of fluorescence likely representing spermatids, Figure 7C). This change in expression from a regular extended punctate pattern (Figure 7B) to a concentrated bilateral arrangement (Figures 7C,D) likely represents the transition into the final stages of spermatogenesis as the worm develops into a young adult. Based on these developmental changes, and the localisation of the developing germ cells, it can be deduced that SPE-15/HUM-3 is expressed in spermatocytes and spermatids at the late stages of spermatogenesis, just before spermatid budding near the spermatheca. SPE-15/HUM-3 expression is apparently downregulated in the gonads post the young adult stage as fluorescence is not found in a spermathecal structure throughout adulthood, supporting a model wherein SPE-15/HUM-3 is here expressed solely in spermatocytes and early spermatids as spermatogenesis is occurring, but not afterwards.

**FIGURE 8:**
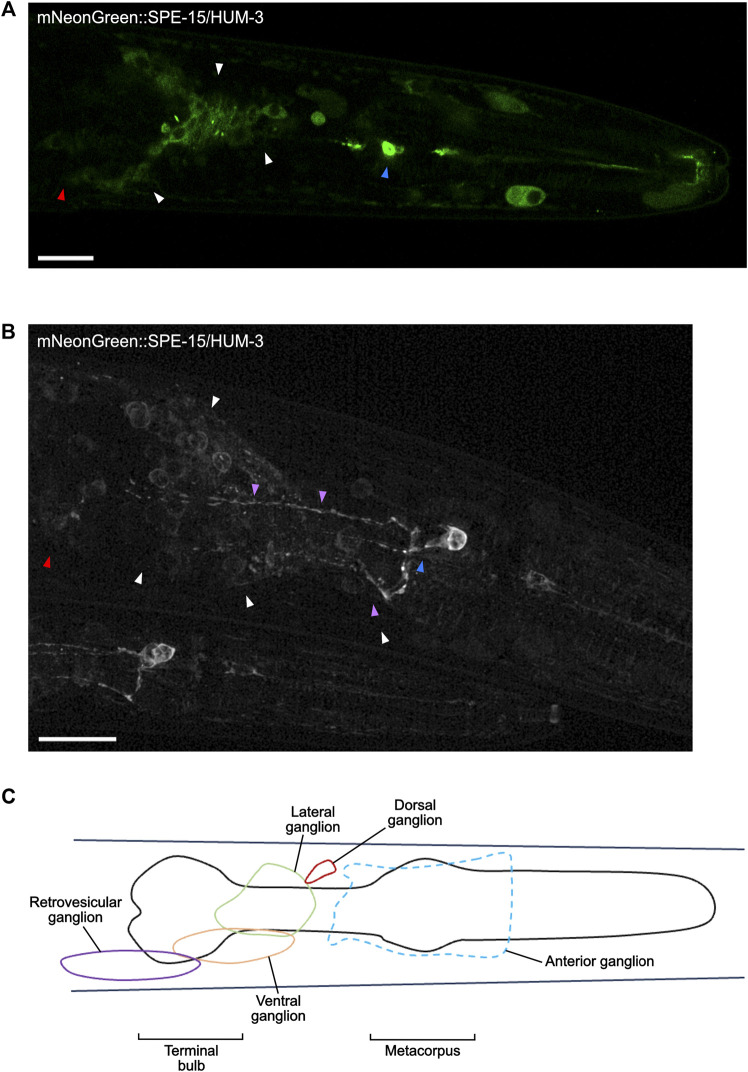
SPE-15/HUM-3 is present in cells of the nervous system within the *C. elegans* head. **(A)** An Airyscan image of SPE-15/HUM-3 expression in the head taken at ×63 magnification. **(B)** A super-resolution image of SPE-15/HUM-3 expression within a *C. elegans* head showing the same region as in **(A)**. Scale bars, 10 µm. Note that a younger worm can be found adjacent to the older, larger one. **(C)** A representative schematic highlighting the main regions, known as ganglia, containing the majority of neuronal cell bodies in the head region.

Super-resolution imaging of the head of mNeonGreen:SPE-15/HUM-3 worms shows SPE-15/HUM-3 concentrating to neuronal cell bodies around the terminal bulb of the pharynx (white arrows, Figures 8A,B, Supplementary Video). This region is occupied by neurons of the lateral and ventral ganglia (Figure 8C), which are primarily composed of interneurons and sensory neurons. SPE-15/HUM-3 is also localised to neuronal cell bodies in the retrovesicular ganglion, which is composed of both motor neurons and interneurons (red arrows, Figures 8A,B, Supplementary Video). Based on collated expression data (Wormbase data release WS282, 2021), it is most likely that SPE-15/HUM-3 localises to interneurons, which typically transmit signals between different neurons. Importantly, the expression of SPE-15/HUM-3 is not restricted to cell bodies; fluorescence is present in long, thin projections going across the pharynx–these projections are likely to be neuronal axons (Figure 8B). SPE-15/HUM-3 is also expressed in a prominent bulbous neuronal cell body in the metacorpus of the pharynx (blue arrows, Figures 8A,B). The WormAtlas database was used to map the identity of this neuron, which suggested it to be NSM/L (NSM indicates neuron class; “L” indicates left, position in worm), a neurosecretory-motor neuron. Interestingly, SPE-15/HUM-3 further localises to an axon with a “dashed” pattern that terminates at the terminal bulb (purple arrows, Figures 8A,B). It is unclear which neuron the process is associated with, but it could possibly be that of NSM/L, whose processes are known to periodically swell, forming structures containing vesicles to which SPE-15/HUM-3 could possibly be localising (Axang et al., 2008).

In summary, our extensive imaging analysis has identified distinct developmental and tissue expression patterns for the MYO6 homologues SPE-15/HUM-3 and HUM-8 in *C. elegans*, supporting the conclusion that both of these myosins of class VI function uniquely and specifically within their respective tissues.

## Discussion

Despite the extensive cellular and biophysical characterisation of human MYO6, the biophysical properties, tissue distribution and function of the two *C. elegans* MYO6 homologues, SPE-15/HUM-3 and HUM-8, are largely unknown. In this study, we confirm that SPE-15/HUM-3 and HUM-8 are indeed minus-end-directed myosins of class VI. These myosins have comparable biophysical and structural characteristics to human MYO6. However, they also possess distinctive structural and sequence-specific elements that set them apart from other myosins of class VI. Through our imaging analysis we provide, for the first time, visualisation of the unique developmental and tissue expression patterns of both myosins endogenously within the nematode. This sheds new light on the potential diversification of MYO6 function in *C. elegans*.

Our sequence and predicted structural analysis of SPE-15/HUM-3 and HUM-8 classifies both motors as myosins of class VI with a high degree of sequence similarity to human MYO6. Key myosin class VI-defining elements, including insert-1, which has been suggested to modulate nucleotide binding, and insert-2 (‘reverse gear’), responsible for the reverse directionality of MYO6, are conserved in SPE-15/HUM-3 and HUM-8. Interestingly, in contrast to human MYO6 both SPE-15/HUM-3 and HUM-8 have N-terminal extensions at their motor domains, which potentially provide additional protein-protein interaction motifs or functional domains. Such examples of myosins include myosins of class III that contain an N-terminal kinase domain (Dose and Burnside, 2000) and MYO16, which contains a 400 amino acid N-terminal extension with several protein interaction motifs. These motifs facilitate binding to other myosin motors. In addition, the extension includes phosphorylation sites and ankyrin repeats which play a role in modifying the ATPase activity (Kengyel et al., 2015). Moreover, studies on class I myosins have demonstrated that the N-terminal region plays a crucial role in refining motor functions, as it helps stabilize the post-power-stroke conformation. This region is a vital structural component in the force sensing capabilities of myosin and indicates a method for creating functional diversity among different myosin isoforms (Greenberg et al., 2015).

Our analysis of the biophysical motor properties of SPE-15/HUM-3 and HUM-8 confirmed them as class VI myosins. *In vitro* motility assays and stopped-flow spectroscopy indicated that SPE-15/HUM-3 and HUM-8 move with similar velocities and display similar ATPase kinetics to human MYO6. Our findings suggest that SPE-15/HUM-3 and HUM-8 share comparable motor properties with human MYO6, emphasising their classification as myosins of class VI. The marginal differences in nucleotide affinities and ADP binding suggest subtle modulations in motor activity but reinforce the overall resemblance to human MYO6.

One key feature of SPE-15/HUM-3 and HUM-8 is the presence of insert-2 in the converter region. This insert repositions the lever arm, which plays a crucial role in determining the direction of myosin movement along actin filaments. We determined the direction of movement of SPE-15/HUM-3 and HUM-8 using a cellular assay whereby each myosin was expressed in RPE cells alongside MYO6+, an engineered plus-end-directed MYO6 that induces the formation of filopodia in cells and accumulates at the tips of such protrusions. We found that neither SPE-15/HUM-3 nor HUM-8 localise to filopodia tips, suggesting that they are not plus-end-directed. While our filopodia tip recruitment assay does not provide direct proof that SPE-15/HUM-3 and HUM-8 move towards the minus end of actin filaments, their almost complete absence from filopodia strongly suggests that SPE-15/HUM-3 and HUM-8, along with human MYO6, are not capable of moving towards the plus end of actin filaments. This supports their classification as reverse motors, and their presumed movement towards the minus ends of actin filaments highlights their unique role in cellular processes. Interestingly, different classes of plus-end directed myosins show differential localisation along filopodia. While the highly processive dimeric MYO5A almost exclusively accumulates at the tips, slower monomeric myosins of class I, MYO1E and HUM-5, reach the tip but are also found along the length of the filopodia. This might reflect the slower velocity of MYO1E and HUM-5 compared to MYO5A, which leads to retrograde movement powered by actin treadmilling. In addition, these myosins of class I are also recruited into filopodia through their lipid binding tail domains. In conclusion, while our cellular assay does not directly prove minus-end-directed movement, the minimal localisation of SPE-15/HUM-3 and HUM-8 in filopodia strongly suggests that these two myosins, like human MYO6, have reverse motor characteristics.

While there is considerable conservation in the motor domain between SPE-15/HUM-3 and HUM-8 and human MYO6 and Jaguar, the tail domains of SPE-15/HUM-3 and HUM-8 exhibit significant sequence divergence, with the exception of a few key binding motifs, such as the MyUb ubiquitin-binding domain, the RRL and the WWY/MWY adaptor binding motif (Berg et al., 2001). This suggests that, although SPE-15/HUM-3 and HUM-8 are assumed to perform similar functions to their mammalian counterpart, notable distinctions in known adaptor-binding regions suggest potential interactions with *C. elegans*-specific binding partners. Orthologues for only three of the known MYO6 adaptor proteins have been identified in worms, providing further evidence for the functional divergence between SPE-15/HUM-3/HUM-8 and human MYO6.

PCR analysis of *C. elegans* cDNA revealed that HUM-8, but not SPE-15/HUM-3, is alternatively spliced. SPE-15/HUM-3 is expressed as a single splice isoform, whereas HUM-8 is expressed as two distinct splice isoforms. The second variant of HUM-8 is alternatively spliced in the CBD and has an additional 14 amino acids inserted just upstream of the MWF motif. In human cells, MYO6 is alternatively spliced in its tail domain to produce four distinct isoforms that are differentially expressed in different cell types. Alternative splicing in the CBD is known to modulate the intracellular targeting and function of human MYO6. The SI isoform of human MYO6 contains a c-Src kinase phosphorylation motif (DYD) required for its function (Tomatis et al., 2013). The LI isoform, on the other hand, produces an additional secondary structure, the regulatory helix (α2-linker), that can sterically alter the interaction of MYO6 with its adaptors. This forms a novel clathrin-binding domain in addition to masking the RRL binding motif, preventing interactions with RRL binding partners (Wollscheid et al., 2016). In contrast, there is currently no information regarding the differential expression or function of HUM-8 isoforms in *C. elegans*. The contribution of the 14 amino acid insert to the potential divergence in cellular functions between the two HUM-8 variants remains uncertain. It is yet to be determined whether this region adds functionally significant secondary structural elements or regulatory phosphorylation motifs. The GTCSWGSS sequence found within the unique HUM-8 insert contains four phosphorylatable sites, three serines and a threonine. Currently, databases lack information about potential phosphorylation sites for SPE-15/HUM-3 and HUM-8. Mapping HUM-8 phosphorylation motifs using phosphoproteomics could therefore provide a starting point in understanding the function of this unique insert. In summary, although alternative splicing does occur in at least one of the *C. elegans* MYO6 orthologues, the site of alternative splicing observed in HUM-8 differs from the pattern observed in human MYO6.

Cargo adaptor proteins play a crucial role in orchestrating the diverse functions of human MYO6 in different cellular pathways. Interestingly, from the numerous binding partners of human MYO6 the same orthologues are expressed in *C. elegans* and *Drosophila*, TOM1L2, GIPC and Dab2. Dab2 facilitates recruitment of human MYO6 to clathrin-coated pits/vesicles at the apical domain in polarized epithelial cells, a role that overlaps with the specific expression of HUM-8 in intestinal epithelial cells. Further work will be required to test whether the *C. elegans* orthologue of Dab2 indeed functions with SPE-15/HUM-3 in enterocytes. The other two potential adaptor proteins expressed in *C. elegans* as well as in *Drosophila* are orthologues of Tom1/L2 and GIPC highlighting a conserved function of SPE-15/HUM-3/8 and Jaguar on early endosomes. Interestingly, when expressing GFP-SPE-15/HUM-3, GFP-HUM-8 or the *Drosophila* Jaguar in mammalian cells, the *Drosophila* orthologue, similarly to human MYO6, was recruited to APPL1-positive early endosomes in the cell periphery, whilst both SPE-15/HUM-3 and HUM-8 were predominantly present as diffuse cytosolic pools and neither was associated with any obvious vesicles or organelles.

Imaging of *C. elegans* strains carrying *mNeonGreen::SPE-15/HUM-3* and *wrmScarlet::hum-8* using confocal microscopy indicated that SPE-15/HUM-3 was developmentally regulated during spermatogenesis in the gonads, but expressed throughout the worm lifespan elsewhere, whereas HUM-8 does not undergo any developmental regulation. Super-resolution microscopy revealed distinct tissue expression patterns of SPE-15/HUM-3 and HUM-8. While the latter is concentrated in intestinal cells of the gut, SPE-15/HUM-3 is enriched in several types of neurons in the head, as well as within the gonads. In the head region, SPE-15/HUM-3 localises to the cell bodies and axons of interneurons in the lateral and retrovesicular ganglia. The *C. elegans* connectome consists of about 87 interneurons, with those in the lateral and ventral ganglia near the terminal bulb belonging to neuron classes AV and AI.

The role of MYO6 in neurons has been investigated in various organisms. In mice, MYO6 is highly expressed throughout the brain and within synapses. It forms a complex with its adaptor SAP97, regulating the trafficking of AMPA receptors to and from the plasma membrane (Wu et al., 2002; Osterweil et al., 2005). Loss of MYO6 leads to impaired synaptic transmission in mice (Yano et al., 2006). Interestingly, AV-type interneurons, known for expressing several AMPA receptor subunits, do not express the SAP97 homologue, DLG-1, highlighting the importance of searching for novel MYO6 adaptors (Firestein and Rongo, 2001). Furthermore, in *Drosophila*, the absence of functional Jaguar results in locomotor defects due to improper synaptic vesicle localisation and disruptions in synaptic transmission at neuromuscular junctions (Kisiel et al., 2011). AV interneurons, specifically involved in initiating both forward and backward movements, play a crucial role in locomotion (Piggott et al., 2011). Another significant neuron, NSM/L, where SPE-15/HUM-3 is localised, is also involved in locomotion (Altun et al., 2021). The consistent enrichment of SPE-15/HUM-3 in these interneurons suggests a conserved role of MYO6 in locomotion in *C. elegans*. The localisation of SPE-15/HUM-3 in neuronal cell bodies and axons strongly implies its potential involvement in neuronal endocytosis and mediation of synaptic transmission.

SPE-15/HUM-3 also localises to late spermatocytes in the gonad during spermatogenesis throughout the L4 and early young adult stages. This is in accordance with the established function of SPE-15/HUM-3 in the segregation of cellular components, and the mediation of membrane constriction, during spermatid budding in secondary spermatocytes (Kelleher et al., 2000; Hu et al., 2019). GIPC, the worm homologue of human GIPC-1/-2, has recently been shown to be expressed exclusively in sperm throughout *C. elegans* development, where it appears to function solely in the segregation of cellular components (Kim et al., 2021). The observation of SPE-15/HUM-3 in the gonads here confirms the importance of MYO6 during spermatogenesis in *C. elegans*. It will be crucial to demonstrate a direct interaction between GIPC and SPE-15/HUM-3 to further underscore the importance of this interaction network.

HUM-8 expression, on the other hand, was observed almost exclusively in intestinal epithelial enterocytes. The role of MYO6 in polarised epithelial cells containing apical microvilli is well-established. In polarised Caco-2 cells derived from the human small intestine, the LI isoform of MYO6 concentrates at the apical domain, colocalising with clathrin-coated pits and vesicles (Buss et al., 2001). In mice, the lack of functional MYO6 causes defects in endocytosis from the apical plasma membrane in enterocytes, leading to a loss of brush border cell structure and integetry (Ameen and Apodaca, 2007; Hegan et al., 2012). Considering that we have observed a vesicular expression pattern of HUM-8 in enterocytes, and the prediction of HUM-8 binding to clathrin light and heavy chains (STRING interaction database), this suggests a potential conservation of the endocytic function of MYO6 in *C. elegans*.

The results from this study support SPE-15/HUM-3 and HUM-8 as reverse-directed class VI myosins with similar biophysical characteristics to human MYO6. As observed for human MYO6, HUM-8 undergoes differential alternative splicing generating two distinct splice isoforms. Importantly, SPE-15/HUM-3 and HUM-8 show distinct developmental expression patterns and localisations in *C. elegans,* highlighting functional specialisations of these two myosins of class VI in nematodes. Although key sequence motifs important for cargo adaptor binding are conserved between human MYO6 and the *C. elegans* orthologues, SPE-15/HUM-3 and HUM-8 are, in contrast to *Drosophila* Jaguar, not recruited to APPL1 endosomes in human cells. Interestingly, however, most of the *C. elegans* tissues in which SPE-15/HUM-3 and HUM-8 are enriched, and the pathways in which they are predicted to be involved in, correlate with those in which human MYO6 functions. Although SPE-15/HUM-3 and HUM-8 show distinctive properties pointing to their evolutionary diversification within *C. elegans*, some of the work presented here also suggests conservation of endogenous localisations, and potentially functions, between the *C. elegans* and human MYO6s.

## Material and methods

### Bioinformatics

Protein sequences for SPE-15/HUM-3 (SPE-15, accession Q9TZI9), HUM-8 (accession U4PBY2), human MYO6 (accession Q9UM54), and *Drosophila* Jaguar (accession A0A0B4KGX1) were retrieved from UniProtKB. Genomic and cDNA sequences were retrieved from NCBI (*spe-15* ID 171712, *hum-8* ID 176872, *MYO6* ID 4646). MYO6 isoform three and Jaguar isoform B sequences were used.

The AlphaFold2 database was used for structural predictions of full-length SPE-15/HUM-3, HUM-8, and human MYO6 (Jumper et al., 2021). The structures were modeled in PyMOL (Schrodinger and DeLane, 2020). Multiple sequence alignments were generated using Clustal Omega at EMBL-EBI (Sievers et al., 2011). Alignments were visualised and exported using Jalview (Waterhouse et al., 2009).

### Transgenic C.elegans strains

Fluorescently tagged *C. elegans* strains were generated using CRISPR/Cas9 by SunyBiotech. A 924 base pair insertion of mNeonGreen and 3Xflag was added to the N-terminus of the F47G6.4a.1 gene to generate the SPE-15/HUM-3 strain PHX5136 (spe-15 (syb5136) [*mNeonGreen::3xFLAG::spe-15*]). The Hum-8 strain PHX5091 included a 759 base pair insertion of a wrmScarlet and 3Xflag at the N-terminus of the Y66H1A.6a.1 gene (hum-8 (syb5091)[*wrmScarlet::3xFLAG::hum-8*]). Both strains allow expression of SPE-15/HUM-3 and HUM-8 at endogenous levels.

### Nematode maintenance and age synchronisation

*C. elegans* worms were maintained at 20°C on NGM (nematode growth medium) plates seeded with OP50 (in LB). To synchronise worm developmental stage, the nematodes were washed off the plates using M9 buffer (0.3% KH_2_PO_4_, 0.6% Na_2_HPO_4_, 0.5% NaCl, 1 mM MgSO_4_). Worms were centrifuged at 1,000 rpm for 3 min at 4°C, the supernatant aspirated, and the pellet washed with 10 mL of M9 buffer. This was repeated until the supernatant was clear (typically 3 times). Bleach solution (20% NaOCl, 0.5 M KOH) was added to the pellet, and the tube vortexed for 10 s at 2 min intervals for 10 min. The eggs were centrifuged at 1,000 rpm for 30 s. The supernatant was aspirated, and this step was repeated twice. The egg solution in M9 was then pipetted onto an NGM plate.

### RNA purification from N2 worms and conversion to cDNA

N2 worms grown on 3 × 90 mm NGM plates were washed 3 times with M9 buffer, generating an ∼ 100 μL packed worm pellet. The pellet was resuspended with 1 mL of TRIzol reagent (Invitrogen) and vortexed at 2000 rpm at room temperature in a ThermoMixer F1.5 (Eppendorf). 200 μL of chloroform was added, and the mixture incubated for 15 min at room temperature with occasional vortexing. The mixture was centrifuged at 14,000 rpm for 15 min at 4°C. The supernatant was then transferred to a new microcentrifuge tube, an equal volume of isopropanol added, and the sample incubated for 10 min at room temperature. The sample was then centrifuged at 14,000 rpm for 20 min at 4°C. The pellet was washed with 1 mL of ice-cold 75% ethanol, and centrifuged again at 14,000 rpm for 5 min at 4°C. The supernatant was removed, and the RNA pellet allowed to air dry before being dissolved in 40 μL of RNase-free dH_2_O and incubated at 65°C for 5 min cDNA was produced from purified *C. elegans* RNA using the ProtoScript II First Strand cDNA Synthesis kit (NEB). Reaction mixtures were prepared according to manufacturer’s protocol using Random Primer Mix.

### Molecular biology

All constructs used throughout this study are detailed in Table 1. The human calmodulin used in this study was in a pFastBac1 vector and was a gift from Dr J Sellers, NIH, Washington, United States. Sequences were amplified from the cDNA of unsynchronised N2 worms and subsequently cloned into the appropriate expression vector unless otherwise specified (pFastBac1 for expression in insect cells, all others for expression in mammalian cells).

**TABLE 1:** List of constructs used throughout this study.

Plasmid name	Uniprot accession	Source of template	Primers used (5′-3′)	Encoded protein	References
GFP_SPE-15/HUM-3_pEGFP C1	Q9TZI9	*C. elegans* N2 cDNA	5′-ATA​TCC​GGA​ATG​GAT​AGT​AGC​ACA​CAT​AGT​ACC-3′	GFP-SPE-15/HUM-3	This study
			5′-TAT​GGG​CCC​CTA​TGG​AGT​CCA​CTC​TTG​AAT​TGG3′		
GFP_HUM-5_pEGFP C1	G5ECZ0	*C. elegans* N2 cDNA	5′-AGA​GTC​GAC​ATG​TCG​TAT​GGT​GGA​CAC​GAC-3′	GFP-HUM-5	This study
			5′-ATA​GGT​ACC​TCA​AGC​AAC​TTG​AGC​AGT​CAA​CTG-3′		
GFP_HUM-8_pEGFP C1	U4PBY2	*C. elegans* N2 cDNA	5′-ATAGTCGACATGCTACGAACGTTGAAC-3′ 5′-TATGGTACCCTAATTCAAGCATCCCAATTTCCAATG-3′	GFP-HUM-8	This study
MYO1E_pEGFP C1	Q12965	Human cDNA		GFP-MYO1E	Buss lab
MYO5A_pEGFP C1	Q9Y4I1	Human cDNA		GFP-MYO5A	Buss lab
6xH_SPE-15/HUM-3_1-841_GSG linker C-tag_pFastBac1	Q9TZI9	*C. elegans* N2 cDNA	5′-AAATTTGCGCGCATGCACCATCACCATCACCATGATAGTAGCACACATAGTACC-3′ 5′-AAATTTTCTAGACTACACCCAGGTGTCGATGGAGCCCCTGCCGCTGCCCACCCAGGTGTCGATGGAGCCCCT-3′	6His-SPE-15/HUM-3motor-C-tag	This study
6xH_HUM-8_61-904_GSG-linker C-tag pFastBac1	U4PBY2	*C. elegans* N2 cDNA	5′- AAATTTGTCGACATGCACCATCACCATCACCATATGATAAATGTGTCTCAG-3′ 5′- AAATTTAAGCTTCTACACCCAGGTGTCGATGGAGCCCCTGCCGCTGCCAGCAATTTGTCGTGAGAACCG-3′	6His-HUM-8motor-C-tag	This study
MYO6+_ mCherry C1					Masters and Buss (2017)

### Filopodia localisation assay

RPE cells were grown at 37°C in Dulbecco’s Modified Eagle Medium F-12 Nutrient Mixture (DMEM/F-12 (1:1) (Gibco) supplemented with 10% fetal bovine serum and 1% penicillin-streptomycin solution (Pen-Strep, Sigma-Aldrich). Cells were plated onto glass coverslips and transfected with mCherry-MYO6+ and GFP-myosin constructs using FuGene six according to manufacturer’s protocol. After 24 h, cells were fixed in 4% PFA, permeabilised using 0.2% Triton X-100, and blocked for 1 h in 1% BSA prior to antibody staining. Primary antibodies: anti-GFP (AB_221569, Molecular Probes) and anti-RFP (AB_2336064, ChromoTek). Secondary antibodies: anti-rabbit AlexaFluor^®^ 488 and anti-rat AlexaFluor^®^ 568 (Thermo Fisher Scientific). Additional stains: Phalloidin-647 (Molecular Probes) and Hoechst. Images were taken on Zeiss Axio Imager. Z2 using a Plan-Aprochromat 100x/1.40 M27 oil-immersion objective lens and processed using ImageJ. To quantify myosin localisation, 100 filopodia were counted per coverslip in three independent experiments (n = 3) for each condition.

### Protein purification

ExpiSf9 cells were grown at 2 × 10^6^ cells/mL at 37°C in ExpiSF CD Medium (Gibco) supplemented with 0.4% Normocin (Invivogen). Baculoviral particles and proteins were generated using the ExpiSF expression system (Gibco) according to manufacturer’s instructions. To generate baculovirus particles, 12.5 μg *Bacmid* DNA was mixed with *ExpiFectamine* (Gibco), added to 62.5 × 10^6^ cells, and incubated at 27°C for 72 h. The supernatant containing the viral particles was subsequently harvested and used directly to scale-up expression. 1 mL of baculovirus containing the myosin motor domain and 0.1 mL of baculovirus containing calmodulin (gifted from Dr. J. Sellers, NIH, Washington, USA) were simultaneously added to 200 mL of cells and incubated until cell viability dropped below 60% (approximately 4 days). Cells were pelleted at 500x RCF for 5 min and frozen. For protein purification the cell pellets were resuspended in 10 mL of myosin extraction buffer (10 mM MOPS pH 7.4, 500 mM NaCl, 5 mM MgCl2, 1 mM EGTA, 1 mM DTT) and sonicated for 2 min in 20 s bursts. Extract was centrifuged at 35,000 RPM for 30 min at 4°C. The supernatant was combined with 1 mL of Ni-NTA resin (ThermoFisher) and incubated for 60 min at 4°C. Resin was washed 2x in myosin extraction buffer and 2x with low salt buffer (10 mM MOPS pH 7.4, 0.1 mM EGTA, 100 mM NaCl). Protein was eluted with 4 mL of low salt buffer containing 150 mM imidazole. Fractions were aliquoted and snap frozen in liquid N_2_. Actin was prepared from rabbit muscle as described previously (Spudich and Watt, 1971). The actin was labelled with pyrene at Cys-374. When used at sub-micromolar concentrations, the actin was stabilised by incubation in a 1:1 mixture with phalloidin to prevent depolymerisation.

### Stopped-flow spectroscopy

Stopped-flow experiments were performed as described previously (Walklate et al., 2016) using a HiTech Scientific SF-61DX2 stopped flow spectrometer. Rabbit muscle actin was prepared as previously described (Pardee and Spudich, 1982) and was labelled with pyrene (Sigma) at Cys-374 following the protocol by Criddle et al., 1985. All measurements were performed at 20°C in 20 mM MOPS pH 7.0, 25 mM KCl, 5 mM MgCl2, 1 mM DTT. Fluorescence transients were measured using intrinsic S1 tryptophan fluorescence (excitation at 295 nm, emission through a WG320 filter) or pyrene-labelled actin (excitation 365, emission through a KV389 filter). Data was acquired and analysed in the Kinetic Studio software.

### Motility assays

To measure the velocity of myosin along actin filaments, an *in vitro* motility assay was performed as described in (Aksel et al., 2015; Adhikari et al., 2016). Rabbit muscle actin was prepared as previously described (Pardee and Spudich, 1982). Fluorescent labelling of actin was carried out at a concentration of 50–70 μM G-actin in 5 mM HEPES (pH 7.5), 2 mM MgCl_2_ and 0.1 mM CaCl_2_ with overnight incubation at 0°C in the dark in the presence of 1 M equivalent of phalloidin (Sigma) and 3 M equivalents of tetramethylrhodamine (Molecular Probes). All reagents were dissolved in assay buffer (AB) (25 mM MgCl_2_, 1 mM EGTA, 1 mM dithiothreitol and 25 mM imidazole, pH 7.4) containing 0.1 mg/mL bovine serum albumin (ABBSA), unless otherwise stated. Glass coverslips were coated with 0.2% nitrocellulose and air-dried before use. Reagents were sequentially flowed through the channels in the following order: 50 μL of 4 μM SNAP-PDZ18 (affinity tag), 100 μL of ABBSA to block the surface from nonspecific attachments, 50 μL of 100 nM 8-residue (RGSIDTWV)-tagged myosin, 100 μL of ABBSA to wash any unattached proteins; 50 μL of 30 nM rhodamine-phalloidin-labelled rabbit actin, 100 μL of an oxygen-scavenging system consisting of 5 mg/mL glucose, 0.1 mg/mL glucose oxydase and 0.02 mg/mL catalase and 50 μL 2 mM ATP. All motility experiments were performed at 20°C. Actin filaments were detected using a Zeiss Axio-observer microscope at ×100 magnification. The gliding velocity of filaments was analysed using the Fiji MtrackJ plugin.

### Insert detection

cDNA was amplified using KOD Hot Start DNA polymerase kit (Merck) with the following primers: 5′-GTG​AAA​CGT​CGT​AAC​AAG​GAA-3′ and 5′-ATA​CCA​CAT​TCC​AGA​TTG​AGA-3′ for SPE-15/HUM-3 insert detection, and 5′-GAA​AAG​AAA​CGG​CAA​AAT​GAA-3′ and 5′-GAA​CCA​CAT​ACT​AAC​TTG​ATC​TTT-3’. PCR products were subsequently run on a 1.5% agarose gel. Bands were excised and purified using Qiagen Gel Purification Kit. The DNA was subsequently cloned into a TOPO vector using the Zero Blunt PCR cloning Kit (Invitrogen) for sequencing.

### *C. elegans* microscopy

For live microscopy, nematodes were mounted on a 2% agarose pad and anestheised using 50 mM sodium azide. Confocal images of whole-worms were taken using the Zeiss LSM780 laser scanning microscope with a plan-apochromatic 20x/0.8 M27 objective. Images were acquired in two channels using a plane scan mode: mNeonGreen was excited at 488 nm with a master gain of 837 AU and a pinhole size of 37 μm; wrmScarlet was excited at 561 nm with a master gain of 755AU and a pinhole size of 38 µm. Images were taken using differential interference contrast (DIC) optics. Higher-magnification images of worms were taken using the Zeiss LSM880 laser scanning microscope with a plan-apochromatic 63x/1.40 M27 oil-immersion objective. Images were acquired in two channels, using a plane scan mode: mNeonGreen were excited at 488 nm with a master gain of 837 AU and a pinhole size of 266 μm; wrmScarlet was excited at 561 nm with a master gain of 755 AU and a pinhole size of 300 µm. Airyscan images were acquired using a fixed zoom of 1.8 and a pinhole size of 108 µm. Super-resolution images were taken using the Zeiss Elyra 7 with lattice SIM2. All images were processed with Fiji software.

## Data Availability

The original contributions presented in the study are included in the article/Supplementary Material, further inquiries can be directed to the corresponding author.
