# Supplementary figures and images for: The two C. elegans class VI myosins, SPE-15/HUM-3 and HUM-8, share similar motor properties, but have distinct developmental and tissue expression patterns
Source: Front Physiol. 2024 Apr 10;15:1368054. doi: 10.3389/fphys.2024.1368054 (PMC11040104; doi:10.3389/fphys.2024.1368054)

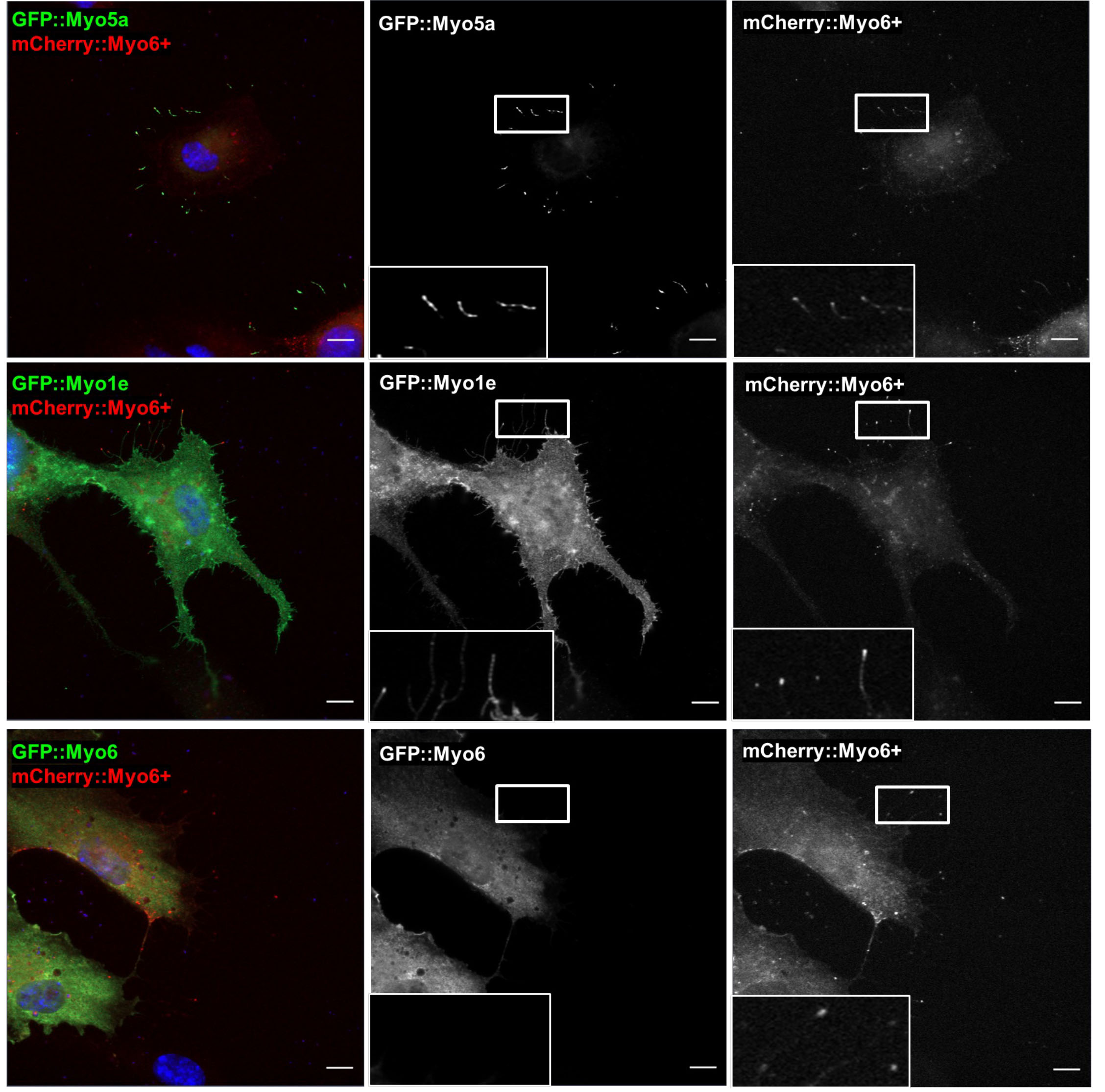

Supplement: Supplementary file 1 [file Image1.JPEG]
