# Supplementary material for: The two C. elegans class VI myosins, SPE-15/HUM-3 and HUM-8, share similar motor properties, but have distinct developmental and tissue expression patterns
Source: Front Physiol. 2024 Apr 10;15:1368054. doi: 10.3389/fphys.2024.1368054 (PMC11040104; doi:10.3389/fphys.2024.1368054)
Supplement: Supplementary file 2 [file DataSheet1.PDF]

A

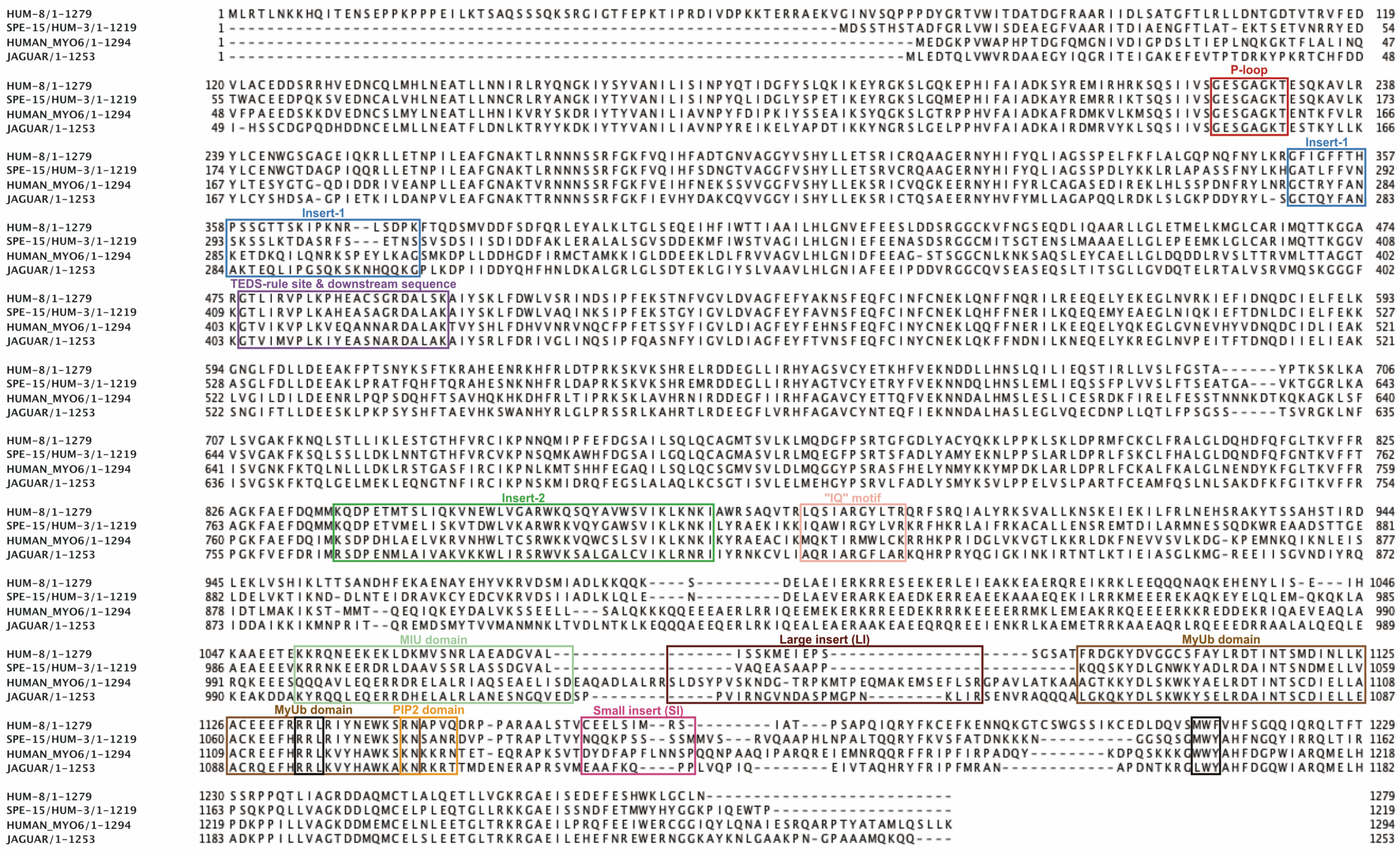

B

	Human MYO6	Jaguar	SPE-15/HUM-3	HUM-8
Human MYO6				
Jaguar	51.2%			
SPE-15/HUM-3	48.1%	43.5%		
HUM-8	45.4%	38.7%	63.3%	
